# Anti-TLR4 biological response to titanium nitride-coated dental implants: anti-inflammatory response and extracellular matrix synthesis

**DOI:** 10.3389/fbioe.2023.1266799

**Published:** 2023-12-05

**Authors:** Stefano Oliva, Francesca Diomede, Ylenia Della Rocca, Antonella Mazzone, Guya Diletta Marconi, Jacopo Pizzicannella, Oriana Trubiani, Giovanna Murmura

**Affiliations:** ^1^ Department of Innovative Technologies in Medicine and Dentistry, University “G. d’Annunzio” Chieti-Pescara, Chieti, Italy; ^2^ Department of Engineering and Geology, University “G. d’ Annunzio” Chieti-Pescara, Pescara, Italy

**Keywords:** oral stem cells, extracellular matrix, dental implants, inflammation, surface topography, titanium

## Abstract

Osteointegration is a key process during dental implant placement and is related to titanium surface topography. Implant coating and surface modification methods ameliorate the bone production and the osteogenic process. The current work aimed at evaluating the biological outcomes of two different surfaces of dental implants, machined and titanium nitride (TiN) coated, at an inflammation level using an *in vitro* model of human periodontal ligament stem cells. The TLR4/MyD88/NF-κB p65/NLRP3 pathway induced by the *Porphyromonas gingivalis* lipopolysaccharide was studied by means of gene- and protein-level expression. Moreover, the expression of vimentin, vinculin, and fibronectin was evaluated to investigate their effects on the cell adhesion and extracellular matrix deposition. The results of the present study suggest that TiN-coated titanium disks may modulate inflammation by the suppression of the TLR4/MyD88/NF-κB p65/NLRP3 pathway and accelerate extracellular matrix apposition.

## 1 Introduction

Titanium implants have demonstrated a considerable clinical success as they allow high integration between the implant surface and the host bone tissue, i.e., osteointegration. The structural properties and constitution of the extracellular matrix (ECM) are related to the differentiation capacity of cell survival and cell activity. Any alteration in the ECM may significantly modify the homeostasis of the tissues, which may have a strong impact during osteointegration. Several surface modification treatments have been recommended in order to improve osteointegration. A change in the shape and activity of mesenchymal stem cells (MSCs) can be induced through surface nanotopography and microtopography. This determines a greater induction of osteogenic differentiation of these cells through an overexpression of osteoblastic genes (Pellegrini et al., 2018; Marconi et al., 2020).

Dental implant surfaces with a wide variety of surface roughness values can be modified either with smooth machined surfaces or roughened with coatings, abrasion or sandblasting, acid etching, or a combination of multiple procedures (Scarano et al., 2003a; Diomede et al., 2020). Titanium nitride (TiN) has attracted significant interest due to its mechanical characteristics, including corrosion resistance, hardness, and shear strength, and biological characteristics, such as biocompatibility and hemocompatibility (Hendry and Pilliar, 2001). Thin coatings of TiN and TiO_2_ are applied using physical vapor deposition (PVD) techniques that alter the surface chemical makeup and crystalline structure while retaining the surface microroughness of the implant (Ballo et al., 2013). In the atomistic deposition PVD method, the material is vaporized by a solid or liquid source, passed as a vapor through a vacuum or low-pressure gaseous (or plasma) environment, and transferred on the substrate where it condenses (Yeniyol et al., 2013). The pathogenesis of most bacterial illnesses starts with the adhesion of bacteria to the implant surface. Accordingly, preventing bacterial infection in intraoral hard or soft tissue is essential. Surface modification treatments have been reported to reduce bacterial adhesion to the implant surface (Berglundh et al., 1992). Grossner-Schreiber et al. (2001) showed that TiN film-coated titanium displayed fewer bacterial colonies when compared to a machined uncoated titanium surface. Brunello et al. (2018a) found a higher percentage of dead bacteria in TiN-coated titanium disks compared to pure titanium. One of the most prevalent bacteria in the oral cavity is *Porphyromonas gingivalis,* which is involved in plaque biofilm formation. Virulence of this Gram-negative bacterium is often caused by the presence of lipopolysaccharides (LPSs) (Marconi et al., 2021a). *Porphyromonas gingivalis* lipopolysaccharide (LPS-G) is essential for triggering the inflammation and release of proinflammatory cytokines such as interleukin-1 (IL-1), IL-6, and tumor necrosis factor (TNF) (Trubiani et al., 2012a). It is known that LPSs, through the activation of the Toll-like receptor 4 (TLR4), lead to the production of nuclear factor kappa B (NF-κB) and, therefore, proinflammatory cytokines (Trubiani et al., 2012b; Liu et al., 2017a).

TLR4 belongs to the family of TLR receptors which, upon interaction with pathogens, induce a proinflammatory response. One of the activators of TLR4 is the lipopolysaccharide (LPS, endotoxin) from Gram-negative bacteria, leading to the activation of two signaling cascades. The first is stimulated in the plasma membrane and involves the adaptor proteins TIRAP and myeloid differentiation primary response gene 88 (MyD88). The second, through the adaptor proteins TRAM and TRIF, starts in the early endosomes after receptor endocytosis (Ciesielska et al., 2021). Titanium dental implants placed in the bone tissue start a series of molecular and cellular processes that guide the formation of the ECM and the apposition of newly formed bone. One of the fundamental steps for osteogenesis and for osseointegration to take place is the secretion of the components of the ECM, of which fibronectin, laminin, actin, and vimentin are fundamental markers for initiating and improving osteointegration (Marconi et al., 2021b). The ECM molecules, being involved in the cell–material interaction, regulate the cell adhesion to the biomaterial.

The present work aims to study, using an *in vitro* model of human periodontal ligament stem cells (hPDLSCs), the biological outcomes of two different surfaces of dental implants, machined and coated with TiN in the inflammatory pathway TLR4/MyD88/NF-κB p65/NLRP3 activated by LPS-G.

## 2 Materials and methods

### 2.1 Titanium disk

In this study, 50 machined (control) and 50 TiN-coated (test) titanium disks from Resista (Omegna, VB, Italy) were used. The disks were made of grade IV commercially pure titanium and had a circumference of 5 mm and thickness of 2 mm. The disks were automatically cleaned in a cavitation washer using detergents designed to eliminate processing grease and finish purification washes. After the decontamination process in a cold argon plasma reactor in a clean room, the disks were packaged in thermally sealed autoclaving trade bags. Gammatom S.r.l. (Italy) externally sterilized the object using 35 KGy of gamma radiation. PVD coating was used to coat 50 Ti disks with TiN.

The coating had a mean thickness of 1.6 ± 0.2 μm. The coating did not affect the inherent texture of the Ti surface. The surface morphologies of the disks were characterized using a scanning electron microscope (SEM) (Jeol, JSM 5410, Tokyo, Japan), at a voltage of 10 kV.

### 2.2 Ethics statement

The Medical Ethics Committee at the Medical School, “G. d’Annunzio” University, Chieti, Italy (n° 266/17.04.14), approved the procedure and obtained informed consent from human periodontal ligament biopsy donors. Before any subjects’ samples were collected, all subjects formally consented to the experiment. The Laboratory of Stem Cells and Regenerative Medicine and the Department of Medical, Oral, and Biotechnological Sciences are certified in accordance with the quality standard ISO 9001:2008 (certificate no. 32031/15/S).

### 2.3 Cell culture

Five human premolar teeth were obtained from generally healthy subjects (between the ages of 18 and 25 years) for human periodontal ligament biopsy by root scaling using Gracey’s curettes. The tissues were cleaned five times with Dulbecco’s phosphate buffered saline (DPBS) without calcium and magnesium (Euroclone, Cat# ECB4004L) and then cultured using a TheraPEAK™ MSCGM™ (Mesenchymal Stem Cell Growth Medium) BulletKit™ (Lonza, Cat# BEBP18-936), which contains the serum-free TheraPEAK^TM^ MSCGM™ Mesenchymal Stem Cell Growth Medium and TheraPEAK^TM^ MSCGM Supplement. After 15 days, the tissues began to release cells after the media had been replaced twice a week. Cells were trypsinized once they reached 80% confluency to obtain subcultures up to passage 4 (P4) (Marconi et al., 2021c).

### 2.4 Experimental study design

The hPDLSCs were used to conduct the three experimental points stated in the following research design. LPS prepared from *P. gingivalis* was applied as a stimulant to the cells (InvivoGen, Cat# tlrl-ppglps).

- hPDLSCs cultured with a machined disk for 24 h
- hPDLSCs cultured with a machined disk and stimulated with 5 μg/mL LPS-G
- hPDLSCs cultured with a machined and TiN-coated disk for 24 h
- hPDLSCs cultured with a machined and TiN-coated disk and stimulated with 5 μg/mL LPS-G

### 2.5 Confocal laser scanning microscopy

Eight-well culture glass slides (Corning, Glendale, Arizona, United States) with Ti disks were used to cultivate hPDLSCs for 24 h at a density of 6.4 × 10^4^/well. Then, the cells were fixed with 4% paraformaldehyde (ImmunoFix) (Bio-Optica, Cat# 05-K01015) in 0.1 M DPBS for 1 h at RT, washed three times with DPBS, permeabilized with 0.1% Triton X-100 (Sigma-Aldrich Corporation, Cat# T8787) in DPBS for 6 min, blocked for 1 h at RT with 5% of non-fat milk (Euroclone, Cat# EMR100500) in DPBS, and incubated with primary antibodies overnight at 4°C. The primary antibodies used were TLR4 (Santa Cruz Biotechnology, Cat# sc-293072, RRID: AB_10611320), anti-MyD88 (Santa Cruz Biotechnology, Cat# sc-74532, RRID: AB_1126429), anti-NF-κB p65 (Santa Cruz Biotechnology, Cat# sc-8008, RRID: AB_628017), anti-NLRP3 (Santa Cruz Biotechnology, Cat# sc-134306, RRID: AB_2152550), anti-fibronectin (Santa Cruz Biotechnology, Cat# sc-8422, RRID: AB_627598), anti-vimentin (Santa Cruz Biotechnology, Cat# sc-6260, RRID: AB_628437), and anti-vinculin (Santa Cruz Biotechnology, Cat# sc-55465, RRID: AB_630433) at a dilution of 1:200.

The cytoskeleton actin and the nuclei were marked with 1:200 Alexa Fluor 488-phalloidin green fluorescent conjugate (Invitrogen, Cat# A12379) and 1:200 DAPI (Invitrogen, Cat# D3571), respectively, for 1 h at 37°C, followed by incubation with secondary antibody Alexa Fluor 568 red fluorescence-conjugated goat anti-mouse antibody (Invitrogen, Cat# A11031, RRID: AB_144696). LSM800 confocal equipment (Carl Zeiss, RRID: SCR_015963) was used to capture the pictures (RRID: SCR_015963) (Marconi et al., 2021d). Images were collected using an argon laser beam with excitation lines at 488 nm and a helium–neon source at 543 and 633 nm. The acquisition settings were kept constant between specimens. ImageJ software was used to analyze the captured images. The quantification was based on 10 images randomly collected.

### 2.6 Western blotting analysis

The hPDLSC protein lysates were used for the Western blot analysis. After electrophoresis, 50 µg of the lysate was transferred to a PVDF membrane using a semi-dry blotting device (Bio-Rad Laboratories Srl, Cat# 1703940, RRID: SCR_019036). The membranes were first saturated with 5% non-fat milk in DPBS and 0.1% Tween-20 (Sigma-Aldrich, Cat# P1379) and then incubated overnight 4°C with the following primary antibodies: anti-TLR4 (Santa Cruz Biotechnology, Cat# sc-293072), anti-MyD88 (Santa Cruz Biotechnology, Cat# sc-74532), anti-NF-κB p65 (Santa Cruz Biotechnology, Cat# sc-8008), anti-NLRP3 (Santa Cruz, Biotechnology, Cat# sc-134306) anti-fibronectin (Santa Cruz Biotechnology, Cat# sc-8422), anti-vimentin (Santa Cruz Biotechnology, Cat# sc-6260), and anti-vinculin (Santa Cruz Biotechnology, Cat# sc-55465), all at a dilution of 1:500, and β-actin (Santa Cruz Biotechnology, Cat# sc-47778, RRID: AB_2714189) was used as a loading control at a dilution of 1:750. The membranes were then treated for 1 h at RT with the goat anti-mouse peroxidase-conjugated secondary antibody (Bethyl Laboratories, Cat# A90-116P, RRID: AB_67183). Immobilon Crescendo Western HRP substrate (Merck Millipore, Cat# WBLUR0100), the enhanced chemiluminescence exposure (ECL) method, and image documenter Alliance 2.7 (Uvitec, Cat# XD-79.LS/20M) were used to identify the expressed proteins. ECL development and evaluation were performed using UVIband-1D gel investigation (Uvitec) to evaluate the detected signals. The acquired values were adjusted using β-actin (Marconi et al., 2019). Three separate experiments were conducted to perform the Western blotting analysis.

### 2.7 RNA isolation and real-time-PCR analysis

Real-time PCR was used to examine the gene expression of TLR4, MyD88, NF-κB p65, NLRP3, fibronectin, vimentin, and vinculin in three separate biological replicates. The total RNA was extracted using the PureLink RNA Mini Kit (Thermo Fisher Scientific, Cat# 12183018A). According to the technical bulletin, M-MLV Reverse Transcriptase (Sigma-Aldrich, Cat# M1302) was used to reverse-transcribe the total RNA into cDNA for 10 min at 70°C, 50 min at 37°C, and 10 min at 90°C. Real-time PCR was performed using the Mastercycler ep realplex real-time PCR system (Eppendorf, Cat#5345) to determine the mRNA expression levels of TLR4, MyD88, RELA, NLRP3, FN1, VIM, VCL, and beta-2 microglobulin (B2M) (endogenous marker) in hPDLSCs grown on machined disks, hPDLSCs grown on machined disks with LPS-G stimulation, hPDLSCs grown on machined TiN-coated disks, and hPDLSCs grown on machined TiN-coated disks with LPS-G induction. Specifically, PrimeTime™ Predesigned qPCR Assays for TLR4 (Hs.PT.58.38700156.g, Tema Ricerca Srl), RELA (Hs.PT.58.22880470, Tema Ricerca Srl), MyD88 (Hs.PT.58.40428647.g, Tema Ricerca Srl), NLRP3 (Hs.PT.58.39303321, Tema Ricerca Srl), FN1 (Hs.PT.58.40005963, Tema Ricerca Srl), VIM (Hs.PT.58.38906895; Tema Ricerca Srl), VCL (Hs.PT.58.2753988, Tema Ricerca Srl), and the PrimeTime™ Gene Expression Master Mix (Cat# 1055772, Tema Ricerca Srl) were employed in accordance with standard protocols. The beta-2 microglobulin (B2M Hs.PT.58v.18759587, Tema Ricerca Srl) was used as the standard for the results. The amplification protocol consisted of three phases: preincubation at 95°C for 3 min, followed by 40 cycles of 15 s at 95°C, and an annealing phase for 1 min at 60°C. Each gene’s expression levels were estimated using the 2^−ΔΔCT^ method (Marconi et al., 2021b).

### 2.8 Statistical analysis

GraphPad Prism 5 software (GraphPad, San Diego, RRID: SCR_002798) was used to conduct one-way ANOVA and *post hoc* Tukey’s multiple comparisons analysis. Statistical significance was calculated at *p* < 0.05. The comparative 2^−ΔΔCT^ technique was used to analyze the gene expression data.

## 3 Results

### 3.1 Morphological features of the titanium disk surface

**FIGURE 1:**
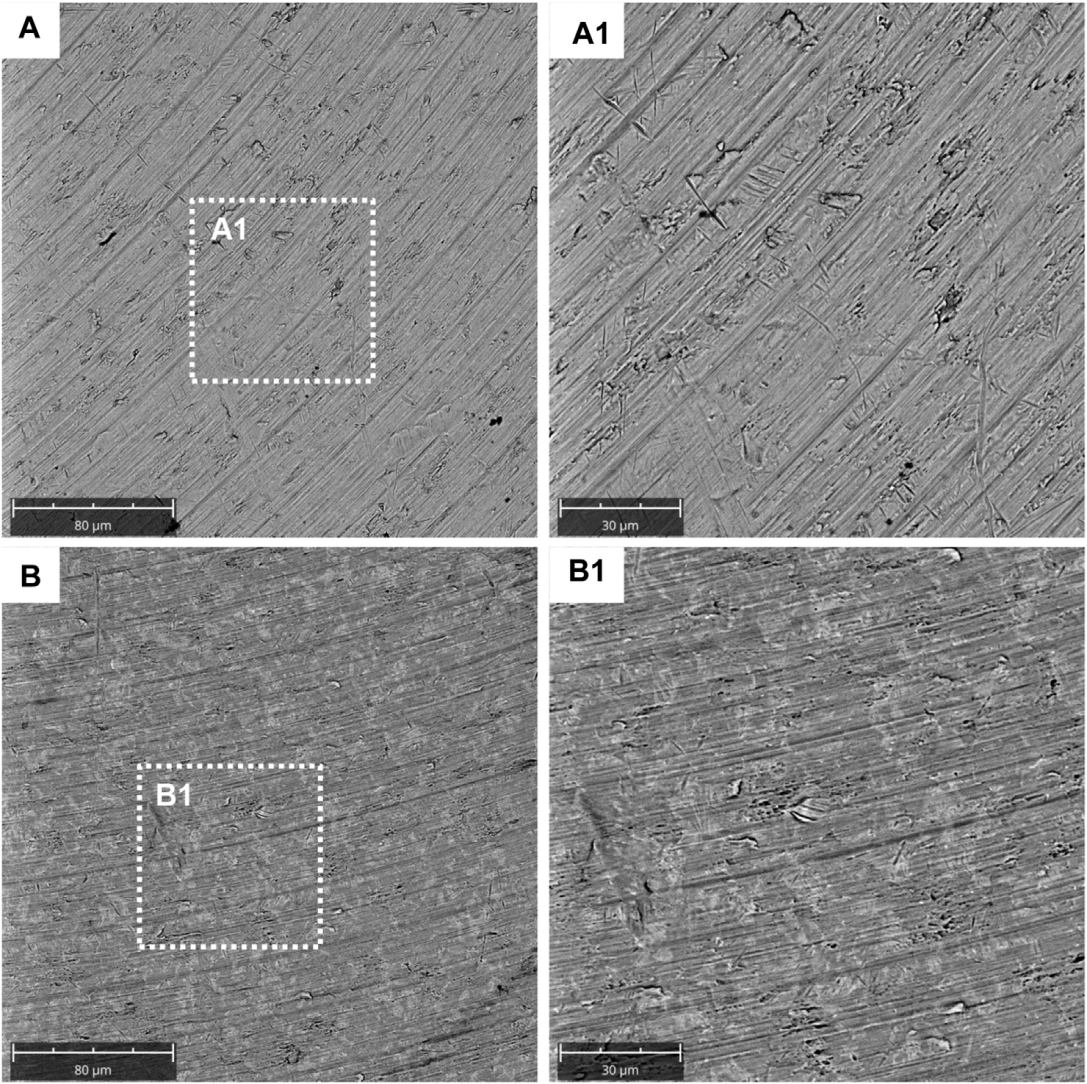
Characterization data for the machined and titanium nitride (TiN)-coated titanium surface. SEM analysis of the titanium machined surface: **(A)** Machined surface disk acquired at 1,000x; **(A1)** machined surface disk acquired at 2,000x; **(B)** TiN surface disk acquired at 1,000x; and **(B1)** TiN surface disk acquired at 2,000x.

SEM observations showed the surface morphology of the two different considered surfaces, machined and TiN-coated titanium surfaces (Figure 1). SEM results showed that the titanium disk surface coated with TiN possesses a rougher surface than the machined disk (Figure A1). At a magnification of 2,000 times of SEM, changes in the titanium surface were observed (Figure B1).

### 3.2 Machined and TiN-coated disk implants influence the inflammatory pathway and the extracellular matrix deposition

**FIGURE 2:**
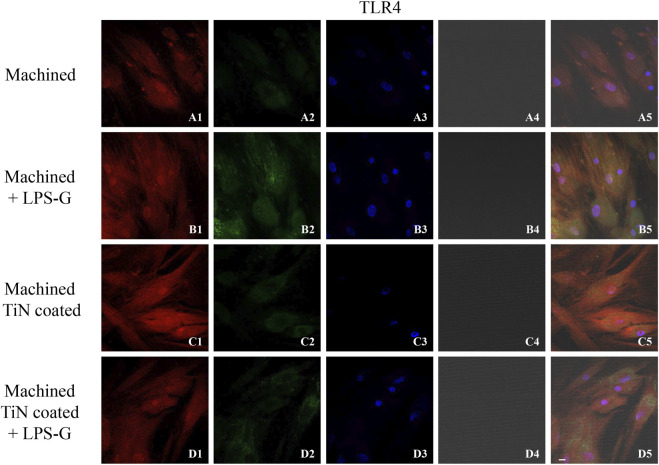
Level expression of TLR4 evaluated by confocal microscopy in hPDLSCs **(A1–D5)**. TLR4 expression in hPDLSCs cultured with the machined disk, in hPDLSCs cultured with the machined disk and induced with LPS-G, in hPDLSCs seeded with the machined TiN-coated disk, and in hPDLSCs cultured with the machined TiN-coated disk and treated with LPS-G. Red fluorescence: TLR4 **(A1–D1)**; green fluorescence: cytoskeleton actin **(A2–D2)**; blue fluorescence: cell nuclei **(A3–D3)**; titanium disk **(A4–D4)**; and merge **(A5–D5)**. Scale bar: 20 μm.

**FIGURE 3:**
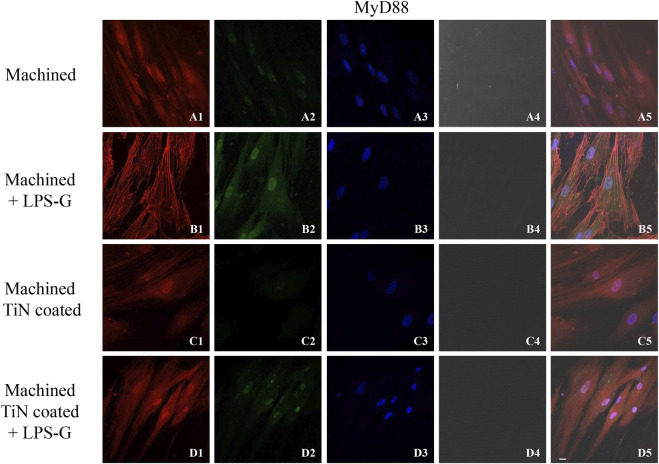
Expression of MyD88 investigated by confocal microscopy in hPDLSCs **(A1–D5)**. MyD88 expression in hPDLSCs cultured with a machined disk, in hPDLSCs cultured with a machined disk and treated with LPS-G, in hPDLSCs seeded with a machined TiN-coated disk, and in hPDLSCs cultured with a machined TiN-coated disk and stimulated with LPS-G: Red fluorescence: MyD88 **(A1–D1)**; green fluorescence: cytoskeleton actin **(A2–D2)**; blue fluorescence: cell nuclei **(A3–D3)**; titanium disk **(A4–D4)**; and merge **(A5–D5)**. Scale bar: 20 μm.

**FIGURE 4:**
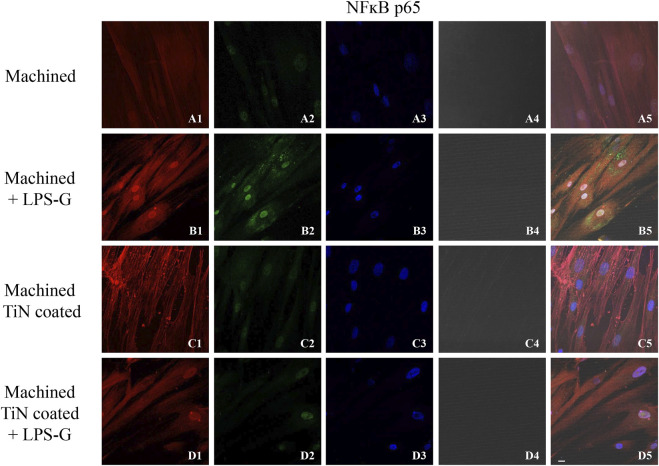
Expression of NF-κB evaluated by confocal microscopy in hPDLSCs **(A1–D4)**. NF-κB expression in hPDLSCs cultured with a machined disk, in hPDLSCs cultured with a machined disk and treated with LPS-G, in hPDLSCs seeded with a machined TiN-coated disk, and in hPDLSCs seeded with a machined TiN-coated disk and stimulated with LPS-G: Red fluorescence: NF-κB p65 **(A1–D1)**; green fluorescence: cytoskeleton actin **(A2–D2)**; blue fluorescence: cell nuclei **(A3–D3)**; titanium disk **(A4–D4)**; and merge **(A5–D5)**. Scale bar: 20 μm.

**FIGURE 5:**
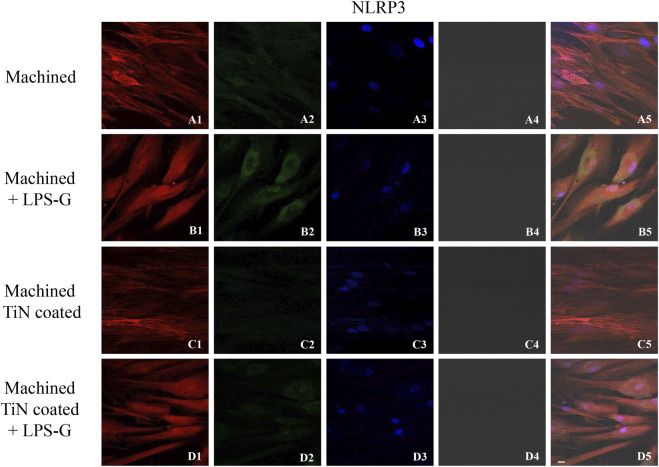
Expression of NLRP3 investigated by confocal microscopy in hPDLSCs **(A1–D4)**. NLRP3 expression in hPDLSCs cultured with a machined disk, in hPDLSCs cultured with a machined disk and treated with LPS-G, in hPDLSCs cultured with a machined TiN-coated disk, and in hPDLSCs seeded with a machined TiN-coated disk and induced with LPS-G: Red fluorescence: NLRP3 **(A1–D1)**; green fluorescence: cytoskeleton actin **(A2–D2)**; blue fluorescence: cell nuclei **(A3–D3)**; titanium disk **(A4–D4)**; and merge **(A5–D5)**. Scale bar: 20 μm.

**FIGURE 6:**
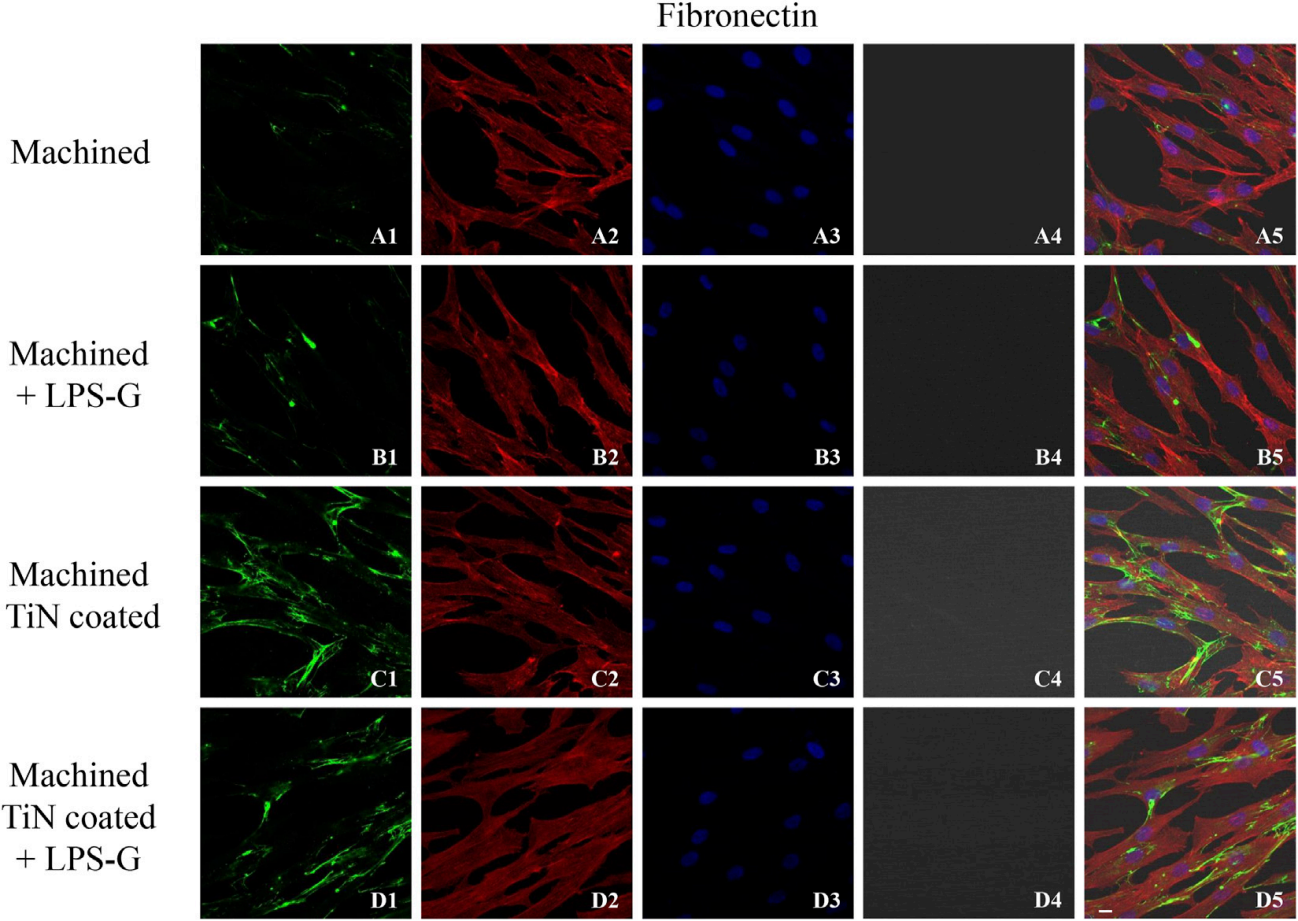
Expression of fibronectin studied by confocal microscopy in hPDLSCs **(A1–D4)**. Fibronectin expression in hPDLSCs cultured with a machined disk, in hPDLSCs seeded with a machined disk and treated with LPS-G, in hPDLSCs cultured with a machined TiN-coated disk, and in hPDLSCs cultured with a machined TiN-coated disk and induced with LPS-G: Red fluorescence: fibronectin **(A1–D1)**; green fluorescence: cytoskeleton actin **(A2–D2)**; blue fluorescence: cell nuclei **(A3–D3)**; titanium disk **(A4–D4)**; and merge **(A5–D5)**. Scale bar: 20 μm.

**FIGURE 7:**
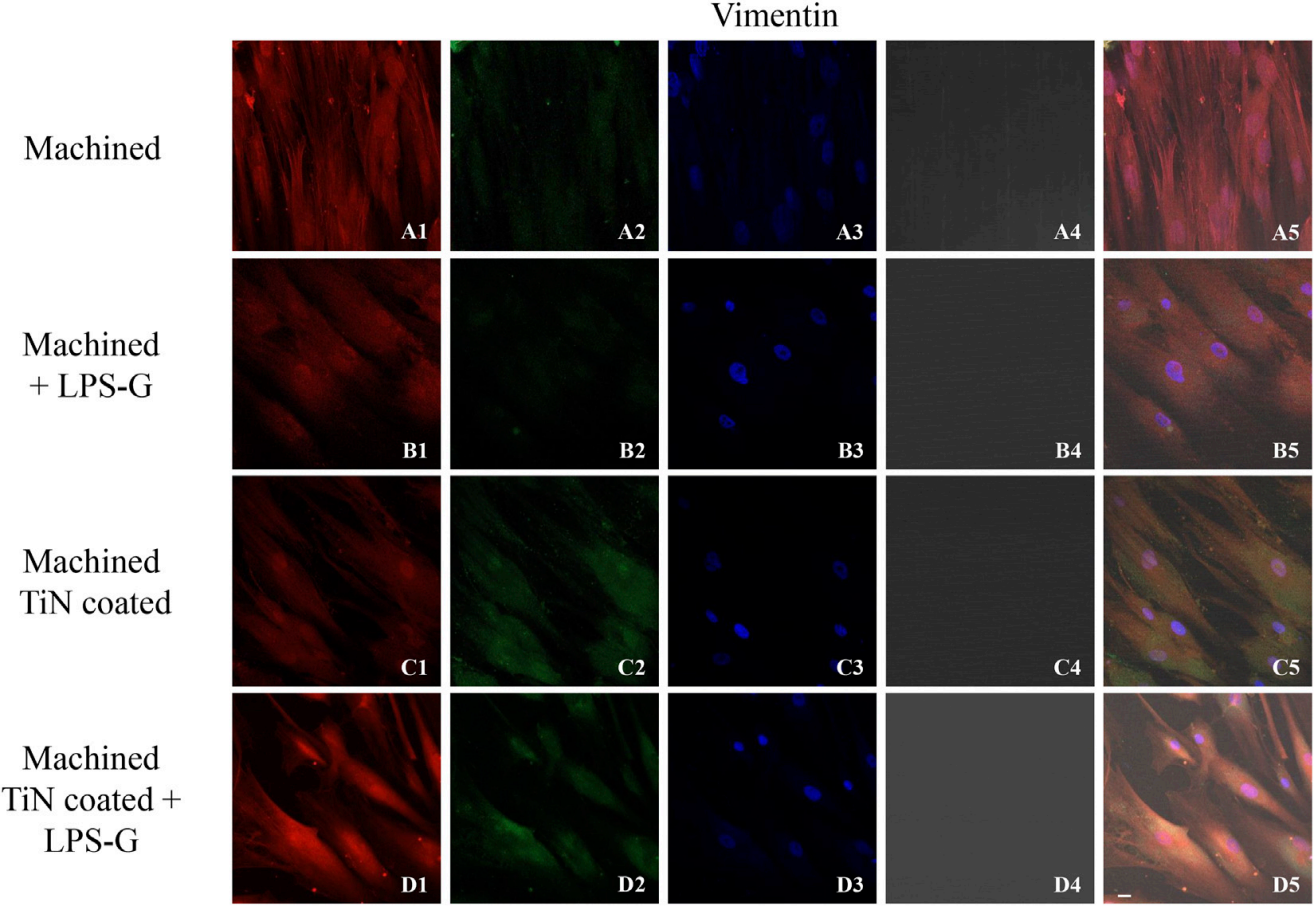
Expression of vinculin evaluated by confocal microscopy in hPDLSCs **(A1–D4)**. Vinculin expression in hPDLSCs cultured with a machined disk, in hPDLSCs cultured with a machined disk and stimulated with LPS-G, in hPDLSCs cultured with a machined TiN-coated disk, and in hPDLSCs cultured with a machined TiN-coated disk and treated with LPS-G: Red fluorescence: vimentin **(A1–D1)**; green fluorescence: cytoskeleton actin **(A2–D2)**; blue fluorescence: cell nuclei **(A3–D3)**; titanium disk **(A4–D4)**; and merge **(A5–D5)**. Scale bar: 20 μm.

**FIGURE 8:**
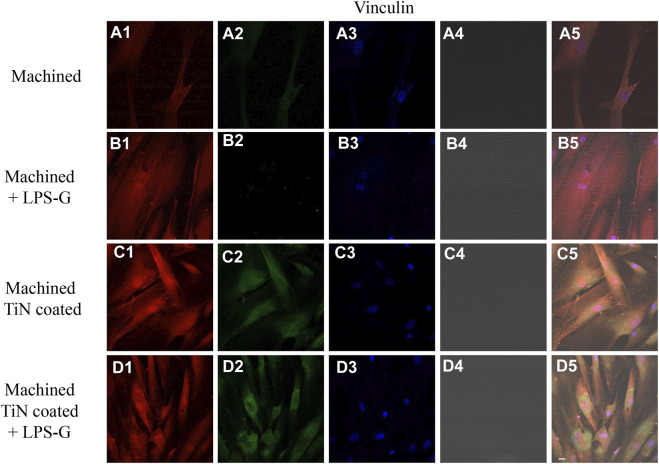
Expression of vinculin examined by confocal microscopy in hPDLSCs **(A1–D4)**. Vinculin expression in hPDLSCs seeded with a machined disk, in hPDLSCs cultured with a machined disk and treated with LPS-G, in hPDLSCs cultured with a machined TiN-coated disk, and in hPDLSCs cultured with a machined TiN-coated disk and induced with LPS-G: Red fluorescence: vinculin **(A1–D1)**; green fluorescence: cytoskeleton actin **(A2–D2)**; blue fluorescence: cell nuclei **(A3–D3)**; titanium disk **(A4–D4)**; and merge **(A5–D5)**. Scale bar: 20 μm.

**FIGURE 9:**
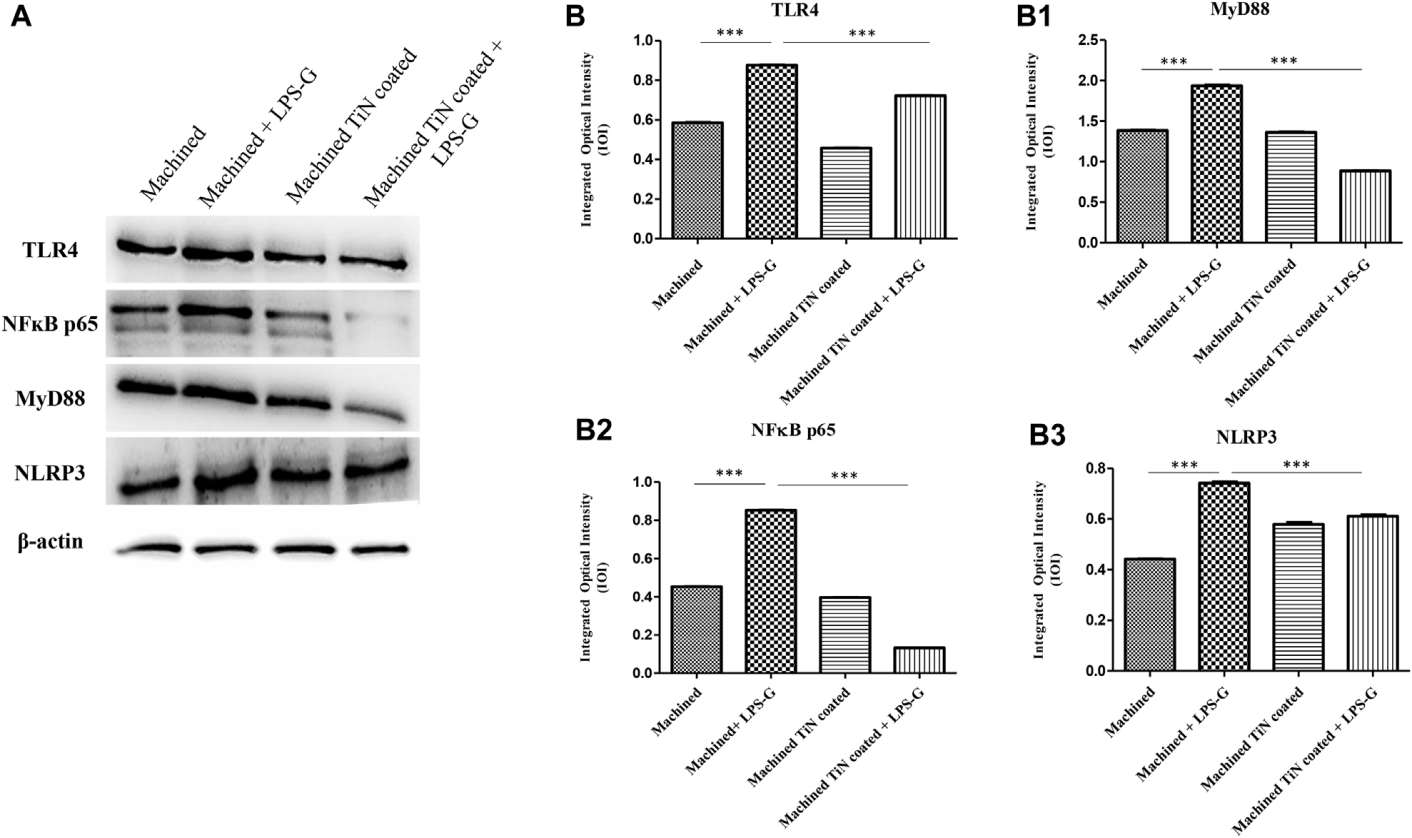
Western blotting analysis. TLR4, MyD88, NF-κB p65, and NLRP3 protein expression in hPDLSCs seeded with a machined disk, in hPDLSCs cultured with a machined disk and treated with LPS-G, in hPDLSCs seeded with a machined TiN-coated disk, and in hPDLSCs cultured with a machined TiN-coated disk and stimulated with LPS-G: **(A)** Each membrane was probed with a β-actin antibody to validate the loading reliability. **(b–b3)** Histograms denote densitometric measurements of protein bands expressed as the mean of the integrated optical intensity (IOI) of three separate experiments. The error bars evidence the standard deviation (±SD). *p < 0.05; **p < 0.01; and ***p < 0.001.

**FIGURE 10:**
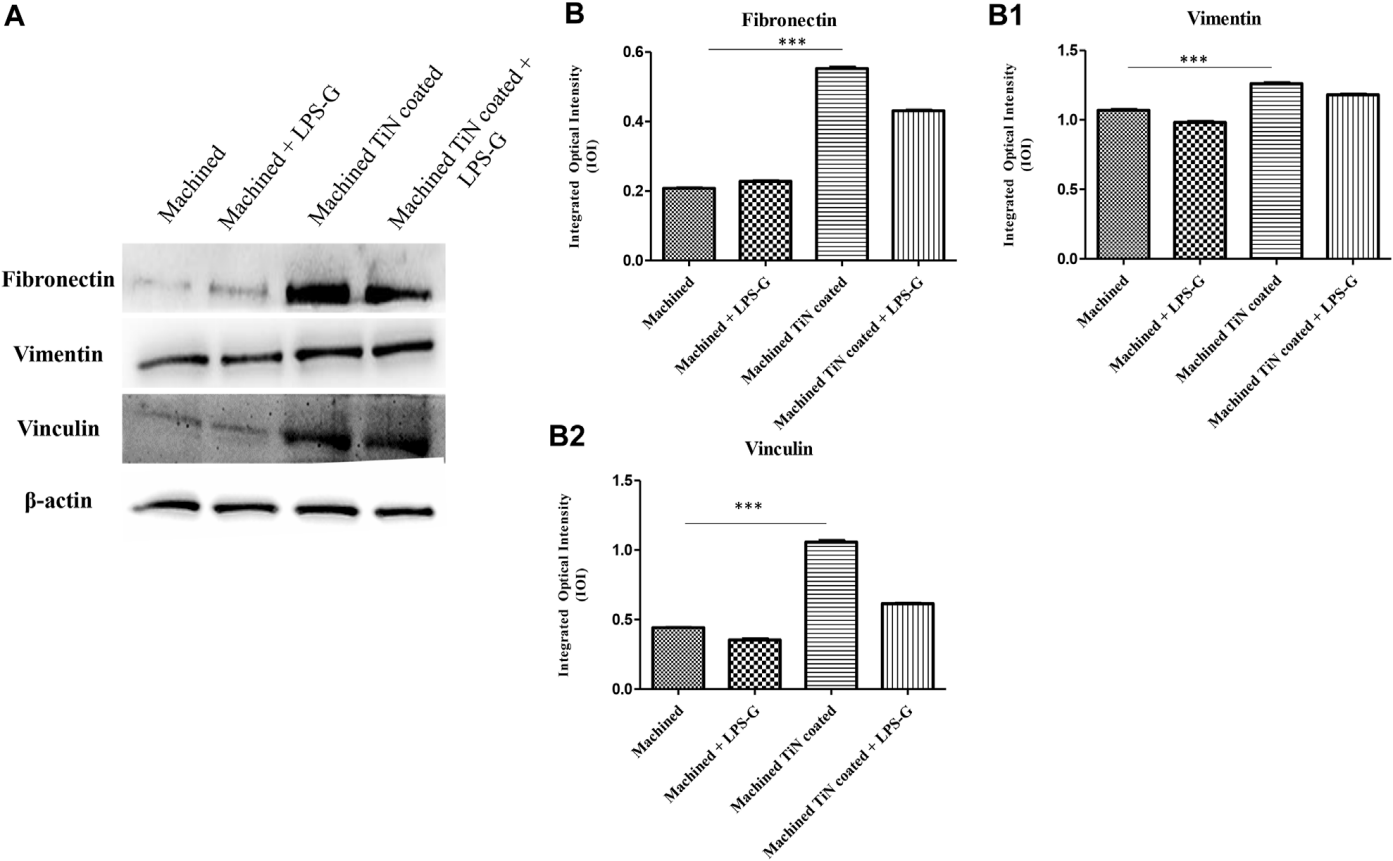
Western blotting analysis. Fibronectin, vimentin, and vinculin protein expression in the hPDLSCs seeded with a machined disk, in hPDLSCs seeded with a machined disk and treated with LPS-G, in hPDLSCs cultured with a machined TiN-coated disk, and in hPDLSCs cultured with a machined TiN-coated disk and induced with LPS-G: **(A)** Each membrane was probed with a β-actin antibody to validate the loading reliability. **(b–b2)** Histograms denote densitometric measurements of proteins bands expressed as the mean of the integrated optical intensity (IOI) of three separate experiments. The error bars evidence the standard deviation (±SD). *p < 0.05; **p < 0.01; and ***p < 0.001.

To study the inflammation response to the LPS-G stimulus, the TLR4/MyD88/NF-κB p65/NLRP3 pathway in the hPDLSC culture was considered. TLR4 is implicated in the immune response; this receptor is activated in the presence of the cell wall component of Gram-negative bacteria, LPS (Ciesielska et al., 2021). TLR4 can activate the MyD88 pathway responsible for the expression of the inflammatory cytokines (Liu et al., 2017a). NF-κB regulates many aspects of innate and adaptive immune functions and acts as a mediator in inflammatory responses. NF-κB induces the expression of various proinflammatory genes, including those encoding cytokines and chemokines, and also participates in the regulation of the NLRP3 inflammasome. The NLRP3 inflammasome is one of the best characterized inflammasomes leading to the production and maturation of IL-1β (Liu et al., 2022).The immunofluorescence observations reported the protein expression of TLR4/MyD88/NF-κB p65/NLRP3 in hPDLSCs cultured with a machined disk implant, hPDLSCs cultured with a machined disk implant stimulated with LPS-G, hPDLSCs cultured with a TiN-coated disk implant, and hPDLSCs cultured with a TiN-coated disk implant stimulated with LPS-G. The obtained representative images showed that the TLR4/MyD88/NF-κB p65/NLRP3 pathway was notably upregulated in hPDLSCs cultured with machined disk implants stimulated with LPS-G for 24 h compared to the hPDLSCs cultured with machined and machined TiN-coated disk implants treated with LPS-G (Figure 2; Figure 3; Figure 4; Figure 5). Furthermore, the protein expression of fibronectin, vimentin, and vinculin was also evaluated by confocal microscopy. The results evidenced that fibronectin, vimentin, and vinculin were upregulated in hPDLSCs cultured with the machined TiN-coated disk implant untreated and stimulated with LPS-G compared with control samples, cells cultured with the machined disk (Figure 6; Figure 7; Figure 8), as also demonstrated by quantitative analysis represented as the arbitrary unit of fluorescence per cell surface unit (F/μm^2^) (Supplementary Figure S1). Western blot analyses provided the protein quantitative expression in order to validate the immunofluorescence acquisition results (Figure 9; Figure 10).

### 3.3 Gene expression

**FIGURE 11:**
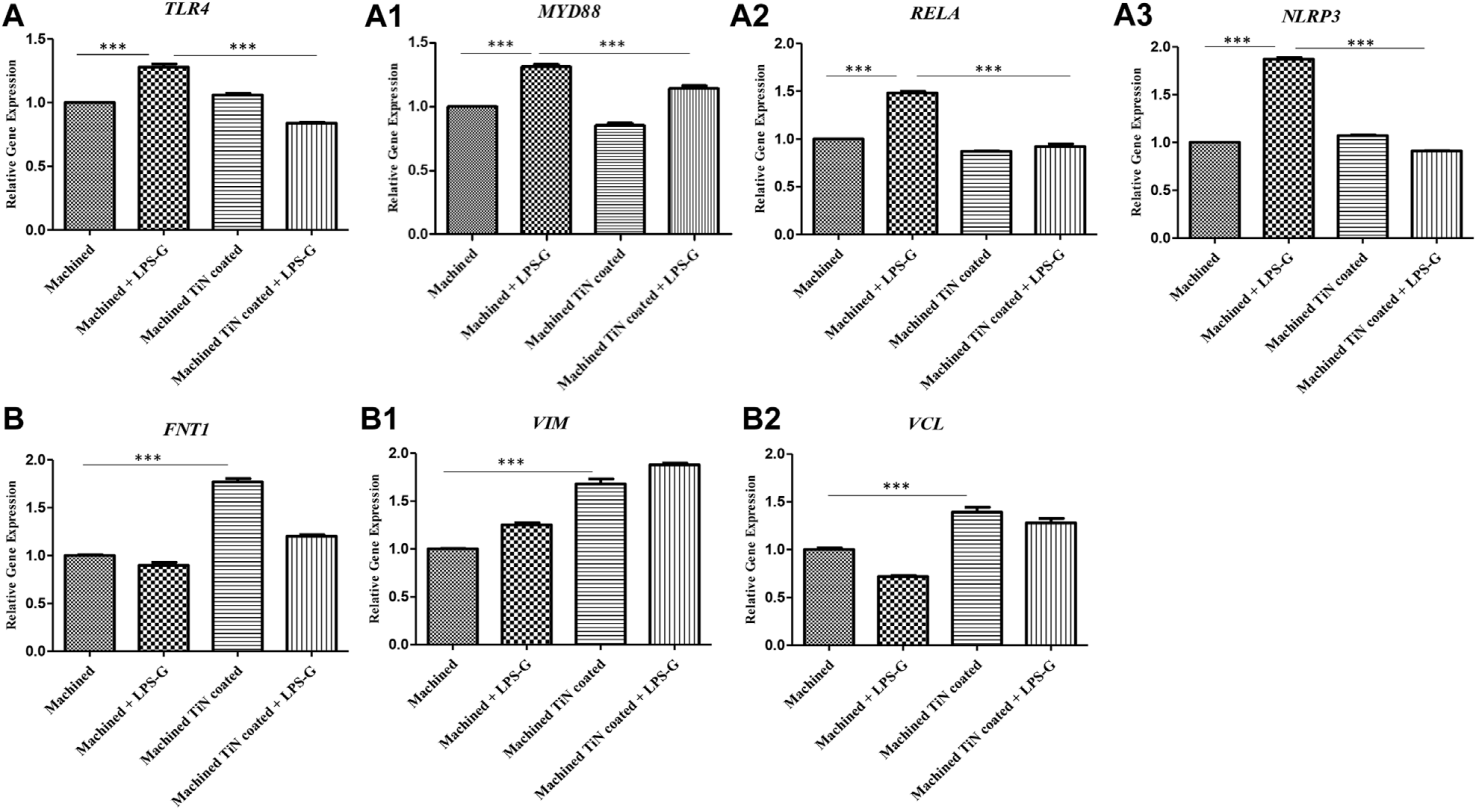
**(a–a3)** Histograms of RT-PCR showing the mRNA levels of TLR4, MYD88, RELA, and NLRP3. **(b–b2)** FNT1, VIM, and VCL in the hPDLSCs cultured with a machined disk, in hPDLSCs cultured with a machined disk and stimulated with LPS-G, in hPDLSCs cultured with a machined TiN-coated disk, and in hPDLSCs cultured with a machined TiN-coated disk and stimulated with LPS-G. *p < 0.05; **p < 0.01; and ***p < 0.001.

The bar graph exhibited the gene expression of TLR4/MYD88/RELA/NLRP3 and FN1/VIM/VCL by real-time PCR. The hPDLSCs cultured with the machined disk and stimulated with LPS-G reported a significantly higher gene expression of TLR4/MYD88/RELA and NLRP3 in comparison with the control samples, cells cultured with machined and machined TiN-coated disks and stimulated with LPS-G. Furthermore, FN1/VIM/VCL gene expression is higher in hPDLSCs cultured with the machined TiN-coated disk than in hPDLSCs cultured with the machined disk. The results obtained by RT-PCR validated the protein expression results (Figure 11).

## 4 Discussion

Osseointegration is the crucial first step in the success of dental implants. It can be defined as biological processes that lead to a prompt attachment of bone tissue to the titanium, and the cellular adherence on the implant surfaces is the first step to ensure long-term clinical success (Anselme and Bigerelle, 2005). Cell adhesion influences cell development, proliferation, and differentiation. Biological compounds, including the ECM, cell membrane, and cytoskeleton proteins, may also be present during the adhesion phase. These molecules cooperate to provide signal transduction, which facilitates the activity of transcription factors and subsequently controls gene expression (Anselme, 2000). Topographical alteration to improve cellular responses at the bone–implant interface has been explored in previous works on implant surfaces (Lange et al., 2002). Coating with a suitable implant material is very important for avoiding its failure and to improve its durability and longevity. Innovative methods, such as physical and chemical approaches, are appropriate coating methods to stimulate the implant surfaces relatively to mechanical, biological, and other multifunctional properties (Lange et al., 2002). Coating the titanium alloy implants with titanium nitride by PVD creates a stable layer on the implant surface that alters its chemistry.

PVD is the most commonly used technique of adding TiN to orthopedic implants, and TiN is obtained by reacting pure titanium with nitrogen gas during a vapor phase prior to accumulation (Pappas et al., 1995). A 48 million cycle wear test was conducted. Thin coatings of TiN and TiO^2^ are applied using PVD techniques, allowing the surface chemical makeup and crystalline structure to alter while retaining the microroughness of the implant surface. PVD methods are engaged to produce films with thicknesses that range from a few to thousands of nanometers (Ballo et al., 2013). Earlier studies demonstrated that TiN coating improves fibroblast cell adhesion, differentiation, and proliferation, in addition to decreasing bacterial adherence and biofilm production. The improvement of wear resistance and abrasive stiffness are fundamental characteristics of TiN film coatings to ameliorate the surface possessions of pure titanium (Durual et al., 2011; Zizzari et al., 2015).

The use of TiN hard thin-film coating in dentistry is strictly limited and recent, although it has been applied in the medical field to coat orthopedic implants (hip and knee replacement prostheses) more frequently (Harman et al., 1997). Preliminary reports demonstrated that TiN coating enhances fibroblast cell proliferation, attachment, and differentiation, as well as reduces bacterial adherence and biofilm formation. Scarano et al. demonstrated that the implant surface covered by bacteria on TiN-coated implants was significantly lower than that of control implants (*p* = .0001) (Scarano et al., 2003b). Grossner-Schreiber et al. (2001) examined the influence of titanium nitride coating on bacterial adhesion compared with the pure titanium surface, concluding that “a significant reduction in the number of adherent bacteria was observed on inherently stable titanium hard materials, such as TiN, compared to polished titanium,” with a statistically significant difference (*p* = 0.0036) (Grossner-Schreiber et al., 2001). Brunello et al. (2018a) showed that the percentage of dead bacteria was higher in the biofilms grown on the TiN-coated disks than those grown on the pure titanium disks (Brunello et al., 2018b). Moreover, S. Datta et al. (2018) reported that TiN-coated implants have shown significant cytocompatibility with mouse fibroblast cells. It was also demonstrated that TiN-coated implants increased osteoblast cell proliferation compared with their sample controls, and the antibacterial activity of TiN-coated implants against different strains of *Streptococci* was also proven, which was bactericidal *in vitro* (Chan et al., 2021).

Based on this purpose, in the current study, the biological outcomes of two diverse disk implant surfaces, machined and TiN coated, in an *in vitro* model of hPDLSCs were exploited in the inflammatory event throughout the modulation of the TLR4/MyD88/NF-κB p65/NLRP3 pathway.

The first TLR identified in mammals was TLR4, which is implicated in the immune response. This receptor is activated in the presence of the cell wall component of Gram-negative bacteria, LPS (Hallman et al., 2001). The activation of this pathway represents a physiological response of the organism to the presence of infection. TLR4 can activate the MyD88 pathway capable of mediating the induction of type I interferons (IFNs) and interferon-inducible genes or a dependent MyD88 pathway responsible for the expression of inflammatory cytokines (Duan et al., 2022). In the MyD88-dependent pathway, the activation of TLR4 drives MyD88, composed of a Toll/interleukin-1 receptor (TIR) domain and a death domain (DD), to recruit other molecules.

NF-κB is involved in the transcription of genes implicated in the immune response and inflammation. The activation of NF-κB can occur through a canonical or non-canonical signaling pathway. The canonical signaling pathway is related to the immune response as well as the activation of the TLR4 pathway induced by LPS. The transcription factor NF-κB regulates many aspects of innate and adaptive immune functions and acts as a mediator in inflammatory responses. NF-κB induces the expression of various proinflammatory genes, including those encoding cytokines and chemokines, and also participates in the regulation of the NLRP3 inflammasome. The NLRP3 inflammasome is one of the best characterized inflammasomes leading to the production and maturation of IL-1β (Liu et al., 2017b).

NF-κB is a protein complex that controls DNA transcription, cytokine production, and cell survival. It is found in almost all cell types and is involved in cellular responses to stimuli, such as stress, cytokines, free radicals, heavy metals, ultraviolet radiation, and bacterial or viral antigens (Mulero et al., 2019). In cells not activated by inflammatory stimuli, NF-κB is sequestered in the cytoplasm by inhibitory proteins called nuclear factor kappa-light-polypeptide-inhibitor the in B-cell inhibitor (IκB). Upon stimulation, IκB is phosphorylated by the IκB kinase (IKK) complex, leading to its ubiquitination and degradation by the proteasome (Woronicz et al., 1997). This releases NF-κB, allowing it to translocate to the nucleus, where it can bind to specific DNA sequences and activate transcription of target genes. The NF-κB pathway is associated with many important diseases, such as cancer, autoimmune diseases, chronic inflammation, metabolic disorders, and neurodegenerative diseases. NF-κB plays a critical role in NLRP3 inflammasome activation. Activation of the NLRP3 inflammasome involves a two-signal process: priming and activation. The first signal, or priming signal, increases the transcription of pro-IL-1β and NLRP3 through the activation of NF-κB (Harder et al., 2009).

Our *in vitro* outcomes insinuate that hPDLSCs placed with the machined disk evidence an augmentation of the level expression of the inflammatory mediators TLR4/Myd88/NF-κB p65/NLRP3 when stimulated with LPS-G or without stimulation. Instead, hPDLSC cells cultured with TiN-coated disks in the presence of LPS-G demonstrated a notable decrease in the TLR4/Myd88/NF-κB p65/NLRP3 level expression compared to the cells cultured with the machined disk alone or in the presence of LPS-G. The decrease in the inflammatory mediators detected in hPDLSCs seeded on the TiN-coated disk surface can be liable for reverting the inflammatory event.

In addition, to further sustain the results achieved, the gene expression of the fundamental markers implicated in cell adhesion, such as fibronectin, vimentin, and vinculin, was also evaluated.

Cellular attachment is crucial in cell communication and regulation, and it also plays a pivotal part in the establishment and preservation of tissue. The mechanical connections between a cell and its ECM can effect and regulate cell conduct and regenerative events (Barker, 2011).

Paschoal et al. (2003) found that TiN coatings exhibited no cytotoxicity, irritation, or acute systemic toxicity responses. The biocompatibility of biomaterials is closely related to the behavior of the contacting cells, in particular, cell adhesion. Cell adhesion is fundamental in tissue reaction and impacts cell growth, proliferation, and differentiation and in the primary stability of the implant osseointegration process (Chien et al., 2008). The cellular affinity to the surface relies on the ECM molecules and denotes a vital aspect for the biomaterial progress (Khalili and Ahmad, 2015). The titanium nitride-coated surface maintained the parameters of the titanium surface, and the nitrogen present on the TiN surface may interfere with the adsorbed proteins, as reported by a previous study (Cavalcanti et al., 2014). Manso et al. found that sputtered TiN coatings deposited on implant surfaces induced cell adhesion and proliferation (Manso et al., 2002).

In the present work, an augmentation in the gene expression of the major molecules implicated in cell adhesion events was found in hPDLSCs cultured with TiN-coated disks alone or with LPS-G. Specifically, a remarkable augmentation of FNT1/VIM/VCL transcribing correspondingly for fibronectin, vimentin, and vinculin underlines that the TiN-coated titanium disk encourages cell-to-cell interaction and cell connections with the neighboring context.

Fibronectin is a multidomain glycoprotein existing in body fluids and on cell surfaces with the primary role of connecting the cell to the ECM. Fibronectin has a ligand-binding site for ECM proteins, such as collagen, fibrin, and heparan sulfate proteoglycans. Fibronectin plays a fundamental part in cell attachment, development, migration, and differentiation, in addition to embryonic development and wound healing (Singh et al., 2010).

The contact of fibronectin with integrins, the main receptor proteins composed of α and β subunits, leads to a receptor conformational alteration and its activation. Integrins are bound to fibronectin adsorbed onto the substrate or incorporated into the insoluble matrix and linked to the actin through linker proteins such as vinculin, a membrane-cytoskeletal protein in focal adhesion plaques that is implicated in the link of integrin adhesion molecules to the actin cytoskeleton (Peng et al., 2011).

Vimentin, also known as the fibroblast intermediate filament protein, plays a vital role in stabilizing the intracellular structure. Vimentin interacts with β1 and β3 integrin subunits and is implicated in cell adhesion in a phosphorylation-dependent way. In addition, vimentin also works as a scaffold for other proteins and contributes to the control of cell migration, adhesion, and division across the direct connection with two other main filamentous factors, actin filaments and microtubules (Paulin et al., 2022).

Even though the current *in vitro* study has limitations, promising results were achieved. These outcomes highlighted the anti-inflammatory properties and the ability of TiN-coated disks to ameliorate cell attachment capabilities, suggesting a better performance in terms of cell/surface interaction.

The future perspective of dental implantology aims at designing an implant surface with definite topography. This study evaluates in an *in vitro* model of hPDLSCs, from one side, the inflammatory event through the investigation of the TLR4/MyD88/NF-κB p65/NLRP3 pathway and, from the other side, a comprehension of the interplay among proteins, cells, and the implant surface.

In this short-term *in vitro* study, cell adhesion factors increased significantly with nitride-coated titanium disks compared to the control uncoated titanium surfaces. The TiN-coated titanium surfaces can control the ECM factor issues and revert the inflammatory processes activated by LPS-G, which may represent an improvement in the biological performance of the dental implants.

## Data Availability

The raw data supporting the conclusion of this article will be made available by the authors, without undue reservation.
