# Supplementary figures and images for: Anti-TLR4 biological response to titanium nitride-coated dental implants: anti-inflammatory response and extracellular matrix synthesis
Source: Front Bioeng Biotechnol. 2023 Dec 5;11:1266799. doi: 10.3389/fbioe.2023.1266799 (PMC10728300; doi:10.3389/fbioe.2023.1266799)

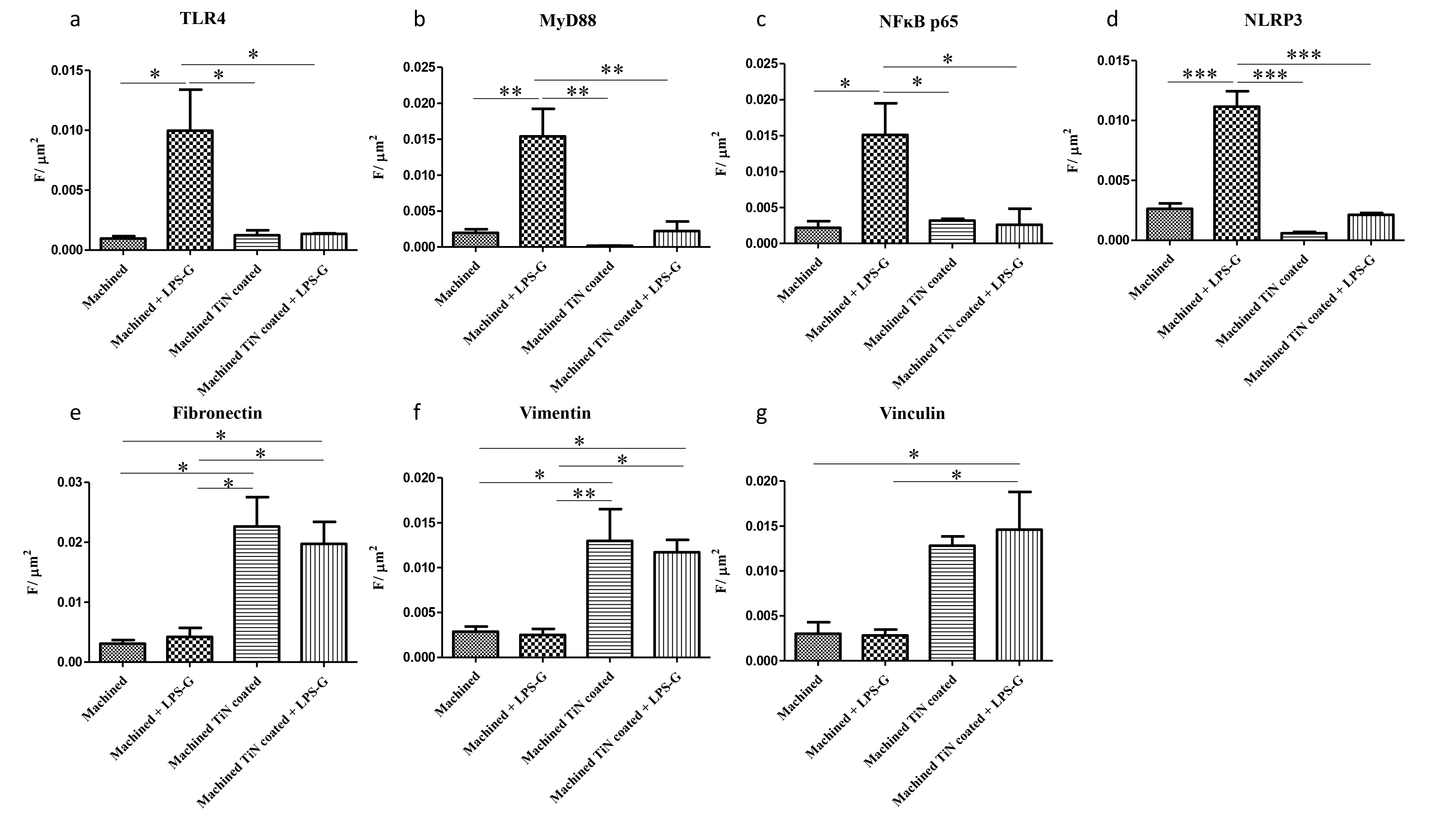

Supplement: Supplementary file 1 [file Image1.tif]
